# PAR-Net: An Enhanced Dual-Stream CNN–ESN Architecture for Human Physical Activity Recognition

**DOI:** 10.3390/s24061908

**Published:** 2024-03-16

**Authors:** Imran Ullah Khan, Jong Weon Lee

**Affiliations:** Mixed Reality and Interaction Lab, Department of Software, Sejong University, Seoul 05006, Republic of Korea; imrankhan@sju.ac.kr

**Keywords:** physical activity recognition, deep learning, machine learning, skeleton data, echo state networks

## Abstract

Physical exercise affects many facets of life, including mental health, social interaction, physical fitness, and illness prevention, among many others. Therefore, several AI-driven techniques have been developed in the literature to recognize human physical activities. However, these techniques fail to adequately learn the temporal and spatial features of the data patterns. Additionally, these techniques are unable to fully comprehend complex activity patterns over different periods, emphasizing the need for enhanced architectures to further increase accuracy by learning spatiotemporal dependencies in the data individually. Therefore, in this work, we develop an attention-enhanced dual-stream network (PAR-Net) for physical activity recognition with the ability to extract both spatial and temporal features simultaneously. The PAR-Net integrates convolutional neural networks (CNNs) and echo state networks (ESNs), followed by a self-attention mechanism for optimal feature selection. The dual-stream feature extraction mechanism enables the PAR-Net to learn spatiotemporal dependencies from actual data. Furthermore, the incorporation of a self-attention mechanism makes a substantial contribution by facilitating targeted attention on significant features, hence enhancing the identification of nuanced activity patterns. The PAR-Net was evaluated on two benchmark physical activity recognition datasets and achieved higher performance by surpassing the baselines comparatively. Additionally, a thorough ablation study was conducted to determine the best optimal model for human physical activity recognition.

## 1. Introduction

Human physical activity recognition (HPAR) has emerged as a crucial area of research with diverse applications in surveillance systems [1], health care systems [2,3], gyms [4], gaming [5], etc. Additional applications of PAR include assisting older people to live freely and safely in the community while utilizing the improved healthcare system. Moreover, vision-based human activity recognition for human–computer interaction (HCI) has attracted the interest of many researchers in recent times. For example, the invention of Kinect enables real-time HCI by recognizing user gestures and body motions to control a character or for gameplay features. However, despite advancements in vision-based HCI, the major obstacle is the automatic recognition of gesture, action, and behavioral context, which is still an open and challenging topic to tackle. Therefore, several researchers utilized Kinect for the evaluation of indoor HPAR for full body tracking and gesture-based detection by an intelligent HCI system [5,6]. Human activity recognition is an essential domain that involves analyzing the gestures and actions that humans perform throughout their daily lives. Researchers use many data modalities to understand and identify these activities, with each modality providing distinct insights into patterns of human motion [7,8,9,10]. RGB video data are one of the modalities that collect visual information by using the colors red, green, and blue and provide a comprehensive perspective of the activities performed by humans [11,12]. They offer a comprehensive depiction of applications’ objects and motions, allowing for meticulous examination using image processing and computer vision methodologies. Although RGB data provide extensive visual information, they often pose difficulties in processing caused by variables such as variations in lighting, obstructions, and distracting background elements, all of which may impact the accuracy of identification. On the other hand, skeletal data offer a different and often more sophisticated method for recognizing activities [13]. They simplify human postures into essential joint coordinates, providing an organized depiction of physical motions [14]. This data type offers a streamlined and concentrated perspective, highlighting the fundamental characteristics necessary for identifying actions. Skeleton data provide a stable and robust representation by recording body joint spatial connections and configurations throughout motions [15]. They are less influenced by external variables like lighting conditions or background clutter. By concentrating on the fundamental elements of joint positions and motions, this abstraction simplifies the recognition process, allowing for a more efficient and precise study of human activities. The advantage of skeletal data over RGB data in certain situations stems from their capacity to condense intricate motions into essential joint locations and trajectories, resulting in a more distinct and less distorted depiction of human activities. The simplified representation of skeletal data allows for more efficient analysis and identification, making them a preferred method in situations where exact activity detection is essential, such as in gesture recognition [16], sports analytics [17], or healthcare applications [18] that need precise movement monitoring. While RGB data provide a wider contextual perspective, skeletal data’s concentrated representation is frequently more appropriate and resilient for the recognition and categorization of human physical activity.

Recently, artificial intelligence-based methods, especially deep learning, have achieved outstanding breakthroughs in human activity recognition. Accurate and real-time recognition of human activities provides valuable insights into understanding human behavior, promoting fitness, and enhancing quality of life. Furthermore, advancements in sensor technology and the availability of large-scale annotated datasets have fueled the development of sophisticated activity recognition systems. Among the various modalities used for PAR, skeleton data have gained significant attention due to their effectiveness, low computational cost, and robustness to occlusions and noise. Skeleton data represent human body movements as a series of 2D or 3D joint positions over time, which can be captured using depth sensors, such as Microsoft Kinect, or through pose estimation techniques [19] from 2D images or videos. The inherent spatiotemporal representation of skeleton data enables a concise yet informative representation of human actions, making them an ideal input for activity recognition models.

Deep learning-based methods have demonstrated remarkable performance for HPAR. However, several challenges are associated with the current literature. The baseline methods utilize CNN or recurrent neural network (RNN) variants for HPAR. However, these methods can only extract spatial or temporal patterns from the data, while HPAR data have spatiotemporal dependencies. To overcome this challenge, the recent literature has developed hybrid models; however, these methods utilize stacked layer phenomena, which extract spatial patterns followed by temporal patterns or vice versa. Furthermore, the output of these methods is directly inputted into fully connected layers for classification. To overcome these challenges, we developed a PAR-Net that extracts both spatial and temporal features simultaneously. The PAR-Net integrates a CNN for spatial patterns and an ESN for temporal dependencies for extraction. The output of both streams is then concatenated, followed by a self-attention mechanism for optimal feature selection. Additionally, we investigate the network’s interpretability, providing insights into how it learns to recognize specific activities from the skeleton data. The main contributions of this research are as follows:

1. The PAR-Net introduces a novel dual-stream paradigm tailored for HPAR, which involves the integration of two distinct streams within the network architecture. The first stream utilized a CNN, which is adept at capturing spatial patterns inherent in the data. Simultaneously, the second stream incorporates an ESN, which is uniquely designed to capture temporal dependencies embedded within the activity sequences. By operating these streams in parallel, the PAR-Net inherently preserves both spatial and temporal characteristics crucial for accurate activity recognition, thereby overcoming the limitations posed by prior models that could only capture spatial or temporal aspects separately or in stacked layer phenomena.
2. The PAR-Net incorporates a crucial self-attention mechanism. This mechanism dynamically highlights relevant spatiotemporal features while suppressing noise and irrelevant information. By allowing the network to focus on critical aspects of the data selectively, the self-attention mechanism significantly enhances the discriminative capabilities of the PAR-Net. This integration empowers the network to learn more salient representations, thereby contributing to improved accuracy and robustness in physical activity recognition tasks.
3. This research extensively examines the PAR-Net and other architectures through thorough ablation studies. Each component, including the dual-stream design, self-attention mechanism, CNN–ESN architecture, and other architecture utilization, is systematically evaluated to understand their individual impact before the PAR-Net selection.
4. To gauge the efficacy and superiority of the PAR-Net, comprehensive comparisons are made against the baseline methods commonly used in physical activity recognition. The evaluation encompasses various metrics, including accuracy, robustness, and computational efficiency, comparing the PAR-Net performance against baselines.

The rest of the paper is structured as follows: Section 2 presents a review of related works in human physical activity recognition and discusses existing approaches using skeleton data. Section 3 details the architecture and components of the PAR-Net. Section 4 presents the experimental setup, dataset descriptions, evaluation metrics, and comparative analysis with state-of-the-art methods. Finally, Section 5 concludes the paper and outlines potential directions for future research in this domain.

## 2. Literature Review

Over the past eight years, significant advancements have been made in human physical activity recognition using 2D skeleton data based on advanced neural network architectures. For instance, Shi et al. [20] demonstrated the efficacy of CNNs for skeleton-based action recognition. The authors proposed a method to map 2D joint coordinates to heatmaps, effectively transforming the skeleton data into a format suitable for CNN input. Although lacking temporal modeling, this study laid the foundation for CNN-based approaches in the field. The authors [21] proposed a novel LSTM network called global context-aware attention LSTM (GCA-LSTM), which mainly focuses on useful joints using a global context memory cell. They also introduced a recurrent attention mechanism to increase the attention capability of the network and combined fine-grained and coarse-grained attention in a two-stream framework. This approach achieves state-of-the-art accuracy on five different challenging datasets utilized by researchers for skeleton-based action recognition. Liu et al. [22] proposed a skeleton visualization method for view-invariant human action recognition. The method involves a view-invariant transform based on sequences to eliminate the influence of view variations, visualizing transformed skeletons as color images, and applying visual and motion enhancement methods to enhance local patterns. A CNN-based model is then used to extract informative and discriminative features from the RGB images, and decision-level fusion is applied to generate action class scores. 

Another research study [23] provided a deep learning-inspired ConvNets-based strategy for activity identification utilizing numerous visual signals. It also presented a novel technique for producing skeletal pictures that reflect motion data. RGB, skeletal data, and depth from RGB-D sensors are employed in the method, which is subsequently trained on ConvNets and merged at the outcome level. Ghazal et al. [24] focused on vision-based human activity recognition (HAR) using skeletal data obtained from standard cameras. The approach extracts motion features from the 2D positions of human skeleton joint data through the OpenPose library. The study used supervised machine learning with different classifiers (K-nearest neighbors (KNN), support vector machine (SVM), naive Bayes (NB), linear discriminant, and feed-forward back-propagation neural network) to recognize four activity classes: standing, walking, sitting, and falling. The experimental results showed that the K-nearest neighbors classifier performed the best among the others. This method offers device-independent HAR for various applications like video surveillance, telecare, ambient intelligence, and robot navigation. Experimental results for deep learning methods like [25], where a CNN and long short-term memory (LSTM) have been adopted to obtain spatial-temporal information, show that the score fusion of CNN and LSTM performs better than LSTM, achieving 87.40% accuracy and ranking 1st in the Large-Scale 3D Human Activity Recognition Challenge in Depth Videos. Li et al. [26] developed actional–structural graph convolutional networks (AS-GCNs) that integrate both actional and structural graph convolutions to capture the dynamics of actions and the spatial relationships between joints. The actional graph convolution captures the temporal dependencies between joints, while the structural graph convolution focuses on modeling the skeletal structure. The AS-GCN demonstrated superior performance on challenging action recognition tasks, emphasizing the importance of considering both spatial and temporal aspects. Another study proposed an intelligent HAR-based system [27] that combines image processing and deep learning techniques using human skeleton information and automatically recognizes human daily activities. This approach has proven promising because of its low computation cost and high accuracy outcomes, making it suitable for embedded systems.

However, effective model training is essential for PAR systems to accurately detect human body parts and the required physical activities. Therefore, Nadeem et al. [28] presented an integrated framework that deals with multidimensional features using a fusion of human body part models and quadratic discriminant analysis. Multilevel features are extracted as displacement parameters, representing the spatiotemporal properties of body parts over time. The recognition engine utilizes a maximum-entropy Markov model to process these characteristics. The experimental findings demonstrated that this method outperformed existing techniques in terms of accuracy for body part identification and physical activity recognition. The body part detection accuracy achieved a rate of 90.91% on the sports action dataset provided by the University of Central Florida. In contrast, the accuracy for activity recognition on UCF YouTube action dataset was found to be 89.09% and the accuracy on IM-DailyRGBEvents dataset was 88.26%, respectively. To capture spatial information and temporal variations for action classification, Tasnim et al. [29] offered a spatio-temporal image formation (STIF) approach for 3D skeletal joints. The transfer learning technique was used by utilizing the pre-trained weights of the models, including MobileNetV2, ResNet18, and DenseNet121, and their proposed method. The method outperformed prior works using UTD-MHAD and MSR-Action3D datasets, including STIF representation, attaining performance accuracy of approximately 98.93%, 98.80%, and 99.65% on UTD-MHAD and 96.00%, 97.08%, and 98.75% on MSR-Action3D datasets for each method, respectively. A dual-stream structured graph convolution network (DS-SGCN) was proposed in [30] to address the issue of skeleton-based action recognition. The DS-SGCN integrates the spatiotemporal joint coordinates and appearance contexts of skeletal joints, allowing for a better representation of discrete joints. The module encodes segregated body parts and dynamic interactions in a spatiotemporal sequence. Structured intra-part graphs represent distinct body parts, while inter-part graphs model dynamic interactions across different body parts. The DS-SGCN acquires intrinsic properties and dynamic interactions of human activity by learning on both inter- and intra-part graphs. After integrating spatial context and coordinate cues, a convolution-filtering process captures the temporal dynamics of human movement. The fusion of two streams of graph convolution responses predicts category information about human action end-to-end. The DS-SGCN technique achieved encouraging performance on five benchmark datasets. 

Another method developed in [31] aimed to assess the efficacy of two RNN models, namely the 2BLSTM and 3BGRU, in accurately detecting daily postures and possibly dangerous situations in a home monitoring system. The analysis was conducted using 3D skeletal data obtained from a Kinect V2 device. The RNN models were evaluated using two distinct feature sets: one composed of eight kinematic characteristics that were manually picked using a genetic algorithm, and another composed of 52 ego-centric 3D coordinates for each skeletal joint, together with the subject’s distance from the Kinect V2. In order to enhance the capacity of the 3BGRU model to make generalizations, they used a data augmentation technique to ensure a balanced training dataset and attained the highest level of accuracy of 88%. Cheng et al. [32] proposed a system for recognizing physical exercise from video frames (extracted 3D human skeleton data using VIBE) using deep semantic features and repetitive segmentation algorithms. The system locates and segments the activity into multiple unit actions, improving recognition and time intervals. Experiments on the “NOL-18 Exercise” dataset showed an accuracy of 96.27% with a 0.23% time error. This system could be used in fitness or rehabilitation centers for patient treatment. Another study [33] presented a human tracking model for squat exercises using open-source MediaPipe technology. The model detects and tracks vital body joints, analyzing critical joint motions for abnormal movements. Validated using a squat dataset from ten healthy individuals, the model uses double exponential smoothing to classify movements as normal or abnormal. The model accurately predicted six out of ten subjects, with a mean square error of 56.31% for normal squat and 29.78% mean square error for abnormal squat setting, respectively. This low-cost camera-based squat movement condition detection model effectively detects workout movement abnormalities. Chariar et al. [34] introduced a method for classifying different types of squats and recommending the appropriate version for individuals. The study utilized MediaPipe and a deep learning-based approach to determine whether squatting is performed correctly or incorrectly. A stacked Bidirectional Gated Recurrent Unit (Bi-GRU) with an attention layer was used to consistently and fairly evaluate each user, categorizing squats into seven classes. The model was compared to other advanced models, both with and without the attention layer, and it outperformed the baselines. Recently, Li et al. [35] developed a unique approach to recognizing human skeletal movement in energy-efficient smart homes by fusing several CNNs. Gray value encoding is used in this technique to encode spatiotemporal information for each 3D skeletal sequence. The CNN fusion model uses three SPI and three STSI sequences as model input for skeletal activity recognition, allowing hierarchical learning of spatiotemporal features. Experimental results were carried out on three public datasets, showing that their performance is better than that of state-of-the-art methods. To address the limitations of traditional machine learning and deep learning models for activity recognition, the authors [36] developed a hybrid model that combines CNN and LSTM for activity recognition. They also generated a new dataset containing 12 different classes of human physical activities, collected from 20 participants using the Kinect V2 sensor. Through an extensive study, the CNN-LSTM technique achieved an accuracy of 90.89%, demonstrating its suitability for HAR applications. Furthermore, Luwe et al. [37] presented a hybrid deep learning model called 1D-CNN-BiLSTM for recognizing human activities using data collected from wearable sensors. Using bidirectional long short-term memory (BiLSTM), the model encodes long-range relationships while transforming sensor time series data into high-level representative characteristics. 

Researchers have explored various approaches, including multi-modal fusion, attention mechanisms, visualization networks, and domain adaptation, to enhance the accuracy, interpretability, and generalization capabilities of the models. These advancements contribute to human activity recognition systems for diverse real-world applications using different types of input data, which collectively contribute to developing a robust and reliable system and also provide valuable insights into building more efficient recognition systems. In summary, the literature review highlights the evolution of methodologies in human physical activity recognition using skeleton data. However, several challenges are associated with the current literature. For instance, hybrid models in HPAR often employ sequential approaches, limiting their ability to extract both spatial and temporal features concurrently. This compromises accurate recognition as it fails to preserve the intricate relationship between spatial and temporal characteristics. Furthermore, existing methods lack efficient strategies for feature selection, potentially leading to suboptimal representations and impacting recognition accuracy. To overcome these challenges, the PAR-Net introduces a dual-stream paradigm, integrating a CNN for spatial patterns and an ESN for temporal dependencies. This simultaneous extraction of spatial and temporal features preserves the essential characteristics for accurate activity recognition. Additionally, the PAR-Net incorporates a self-attention mechanism, dynamically highlighting salient spatiotemporal features, thereby enhancing discriminative capabilities. This comprehensive approach ensures optimal feature representation and addresses the limitations of prior methods, leading to superior performance in accuracy, robustness, and computational efficiency for physical activity recognition.

The existing literature predominantly focuses on solo architectures for HPAR, overlooking the challenge posed by datasets containing both spatial and temporal features. Solo models struggle to effectively extract both types of features, hindering their performance. While some hybrid models have been proposed, such as combinations of CNN and RNN variants, they often utilize a stacked layer phenomenon for feature extraction. This approach, where one model learns from the original data while the subsequent model learns from the extracted features, faces difficulties in capturing effective temporal dependencies due to the reduction in data dimensionality after each convolutional layer. Moreover, the outputs of these models, whether solo or hybrid, are directly fed to dense layers without optimal feature selection. Consequently, the accuracy attained is insufficient for real-world implementation. In response to these limitations, we developed a PAR-Net, leveraging a dual-stream architecture to extract spatial and temporal features from raw data. These features are then concatenated and subjected to a self-attention mechanism for optimal feature selection, followed by classification using dense layers. Notably, the PAR-Net achieves higher accuracy compared to baseline models, addressing the shortcomings of existing approaches.

## 3. Proposed Methodology

A PAR-Net was developed to address the inherent challenges in recognizing complex activities from skeleton data. It incorporates various modules, including CNN, ESN, and self-attention, to extract spatial and temporal features effectively, where the integration of the attention module plays a crucial role in feature selection. All these modules are further discussed in the subsequent sections.

### 3.1. Spatial Feature Extraction

The 1D CNN is an effective architecture for extracting spatial patterns from data [38]. Unlike traditional approaches, which are reliant on handcrafted features, a 1D CNN automatically learns hierarchical representations of spatial features, making it capable of discerning complex patterns. Skeleton-based human physical activity recognition data provide a unique perspective on human movements through joints and key point configurations over time. A 1D CNN uses filters over the spatial dimension for feature extraction that gradually abstracts into high-level feature representation. A CNN layer is followed by a max-pooling layer to reduce the computational burden by focusing on the most salient information in feature maps. To finally classify the resulting features, the output is forwarded to fully connected layers. This architecture excels at learning both short-term and long-term dependencies within the data, making it well-suited for discerning nuanced patterns in activities. The processes of the $l^{th}$ convolution operation, max pooling operation, and fully connected layer can be mathematically demonstrated below where Equation (1) can be written as:

(1) $$Y_{ij}^{l}=\sigma \left(\left(\sum_{m=1}^{M}W_{m,j}^{l}\cdot X_{i=m-1,j}^{0}\right)+B_{j}^{l}\right),$$

Here, $B_{j}^{l}$ is the bias term of the $j^{th}$ feature map, W represents the weights, m is the filter, and σ is the activation function (e.g., ReLU).

(2) $$p_{ij}^{l}={max}_{d_{i}=0}^{s}\left(Y_{i*t+di,j}^{l-1}\right)$$

Equation (2) shows the mathematical representation of the max pooling operation, which downsamples the input by selecting the maximum value for the window. Where t is the stride and s is the size of the pooling operation.

(3) $$d_{i}^{l}=\sum_{j=1}W_{ji}^{l-1}\left(\;\sigma \left(X_{i}^{l-1}\right)\right)+B_{j}^{l-1},$$

where $W_{ji}^{l-1}$ is the weights, σ is the activation function, $X_{i}^{l-1}$ is the input data, and $B_{j}^{l-1}$ is the bias term in Equation (3).

### 3.2. Temporal Feature Learning

The ESN is the reservoir computing architecture that has become popular for processing sequential data, which makes it useful for time series analysis and advantageous in physical activity detection compared to other RNN architectures in terms of effectiveness and efficiency. ESNs can be distinguished from the other RNNs by a fixed, randomly generated reservoir of neurons that function as a dynamic memory. The ESNs have a fixed set of parameters and only optimize the output layer weights during training; they are better suited for applications with minimal labeled data because training is less complicated. The intrinsic memory capacity of ESNs is well suited for processing skeleton data. The reservoir of the network extracts temporal features from the sequential input by identifying patterns and relationships. Due to its dynamic properties, the reservoir can accurately simulate the temporal information of human actions and can accurately identify complex activities. To employ ESNs for activity recognition with skeleton data, the input sequences, which describe the spatiotemporal dynamics of human motions, are fed into the reservoir. The input data are converted into a high-dimensional state space by the reservoir. A linear readout layer that maps the high-dimensional representations to the target activity classes is subsequently trained using the reservoir states. Particularly, the only portion of the network that is trained is this readout layer, which streamlines the optimization procedure. ESNs have the benefit of being robust against noise, capturing long-term dependencies, and providing effective training with a small amount of labeled data. Due to the intrinsic memory provided by the fixed reservoir structure, the network can recognize and make use of temporal patterns in the skeletal data. Moreover, ESN training makes it easy, especially in situations where gathering sizable, labeled datasets might be difficult.

**Figure 1:**
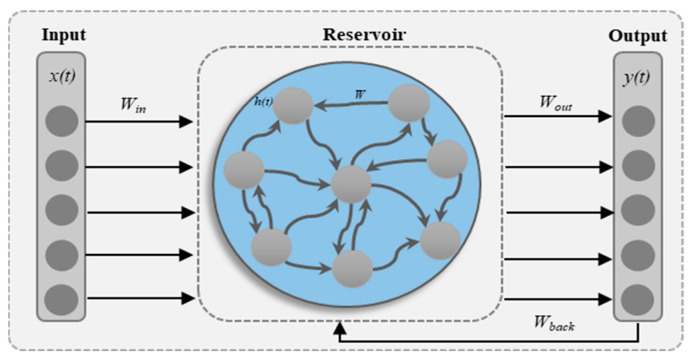
The internal architecture of the ESN.

The ESN is mainly composed of three layers (input layer, reservoir layer, and output layer), as shown in Figure 1. The input layer and reservoir layer are generated randomly. The reservoir layer consists of N neurons and is considered fading memory, where x(t) is the input and h(t) combines it at time t. The state of the ESN is updated by Equation (4), as given in [39].

(4) $$h\left(t\right)=\emptyset (W_{in}x\left(t\right)+Wh\;\left(t-1\right)+W_{back}y\left(t-1\right)+b_{x}),$$

The activation function utilized in reservoir neurons is represented by ∅(◾), the weight matrices of the reservoir layer are represented by $W_{in}\in R^{M\times N}$, the weight matrices of the input and output feedback are represented by $W_{back}\in R^{N\times Y}$, and the input bias is denoted by $b_{x}\in R^{N\times 1}$. The input y(t−1) at the current instant is desired. Hence, $W_{back}y(t-1)$ can be combined with the $W_{in}u(t)$ item. Equation (4) can be written as shown in Equation (5), where $b_{x}$ can be fused into $W_{in}x(t)$ while only connecting the output rather than connecting the input and other neurons in the reservoir layer.

(5) $$h\left(t\right)=\emptyset (W_{in}[1:x(t)]+Wh\;\left(t-1\right)),$$

Equation (6) is used to determine the target *Y*$\left(t\right)$ at time t:

(6) $$Y\left(t\right)=W_{out}h\left(t\right){+b}_{y},$$

where the output weights of the ESN are denoted with $W_{out}\in R^{(N+1)\times Y}$, the output bias is denoted by $b_{y}\in R^{N\times 1}$, and $b_{y}$ is fused with $W_{out}h\left(t\right)$ according to Equation (6), which can be written as shown in Equation (7):

(7) $$Y\left(t\right)=W_{out}[1;h\left(t\right)],$$

ESN implementation has two steps for target prediction, where the first step is specifying $W_{in}$, W, and states of ESN. The input weights $W_{in}$ and W of the reservoir layer are generated randomly and kept constant. The output weights $W_{out}$ are optimized, all states are stored in matrix H, and the targeted outputs are stored in a feature vector $y^{*}$. Finally, the model is connected with a linear regressor where the ridge regressor is used, which computes the regularized least-square problem using Equation (8), where $\beta \in R^{+}$ represents the L_2_ regularization coefficient.

(8) Wout*=argmin W∈R12 ||HW−y*||2+β2||W||2=(HTH+βI)−1HTy*

### 3.3. Self-Attention Mechanism

**Figure 2:**
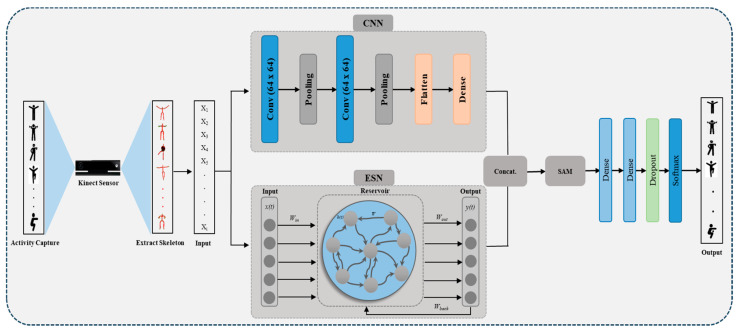
Main framework of the PAR-Net.

The self-attention mechanism is a key tool for improving model performance, particularly in hybrid models. Both stream outputs are combined in order to generate a single feature vector, which is then inputted into SAM to provide a representative pattern for the final prediction. Figure 2 illustrates a visual representation of each stream output, concatenation layer, SAM, and fully connected layer. We integrate the SAM to select optimal features from the data by dynamically adjusting the importance of hidden patterns. SAM is used to identify the key patterns in the merged feature vector of the CNN and ESN streams prior to the prediction process. It also analyzes the correlation of hidden characteristics across different timestamps in each dimension. In SAM, the score of hidden features of the $g^{th}$ time stamp in the $p^{th}$ dimension can be calculated using Equation (9):

(9) $$S_{g,\;p}=f_{sco}\left(W_{g,p}\left[h_{1,p},h_{3,p},h_{3,p},\ldots h_{n,p},\right]\right),\;p=1,2,3\ldots n,\;g=1,2,3\ldots t$$

Here, the $p^{th}$ dimension of the hidden state on the $g^{th}$ timestamp is represented by $h_{g,p}$, the weight metric is indicated by $W_{g,p}$, a function implemented by fully connected layers is represented by $f_{sco}$, the number of timestamps is represented by t, and the hidden feature dimension is represented by n. The final layers of our proposed model are fully connected layers, which are used for activity recognition. The SAM output is flattened to a $Z_{l}=Z_{2},Z_{2},Z_{3}\ldots Z_{n}$ feature vector, where the number of SAM output dimensions is represented by l. The output of the attention module is fed to a dense layer.

### 3.4. PAR-Net

We proposed a dual-stream model with an attention mechanism called PAR-Net to efficiently recognize physical activities using skeleton data. In the spatial stream, CNN layers are employed to extract spatial features from the input skeleton data. These convolutional layers apply filters to the data, capturing hierarchical spatial patterns. In the temporal stream, the ESN is integrated to model the temporal dynamics of the skeletal sequence. In the CNN stream, the data are processed by two 1D-convalutional layers with 64 and 128 filters and a kernel size of 3 and 1, respectively. This process is followed by a dense layer with 128 units and a flattened layer. Meanwhile, the ESN stream utilizes an Echo State RNN cell consisting of 32 units, which has been configured with a hyperbolic tangent (tanh) activation function. The cell is started with predetermined parameters, including decay, epsilon, and alpha, and is configured to update certain variables throughout the training process. The output of both streams is then combined by a concatenation method and forwarded to a self-attention mechanism. A self-attention mechanism is used to select more prominent features from the combined feature vector. These optimal features are then fed to a dense layer consisting of 64 units using a rectified linear unit (ReLU) activation function. Finally, a SoftMax layer consisting of 5 units (depending on the number of classes) is applied to calculate the model output probabilities for the various activity classes. The model is trained using the Adam optimizer, with a learning rate of 0.0001, and the categorical cross-entropy loss function. During the training process, the model is adjusted to the training data using a batch size of 32 for 100 epochs.

## 4. Experimental Results

This section delves into a detailed exploration of the evaluation metrics, datasets, and performance comparisons of different models. Specifically, we focus on assessing the efficacy of the PAR-Net in comparison to baseline models. We have used two datasets for the evaluations of our proposed model and the dataset details are given in Table 1. Several evaluation metrics are used to assess the performance of models that aim to recognize or classify human activities based on skeletal joint data captured from devices. We used accuracy, precision, recall, and F1-score for performance and comparative analysis; the details of these metrics are given in [36]. The experiments are conducted on a computational setup composed of a GeForce RTX 3070 GPU with 8 GB of RAM, a Core i7 processor with 32 GB of onboard memory, and the Windows 10 operating system (Nvidia Corporation, Santa Clara, CA, USA). The implementation was carried out using Python V3.7.4, employing the Keras deep learning framework built on TensorFlow. 

### 4.1. Dataset Description

**Figure 3:**
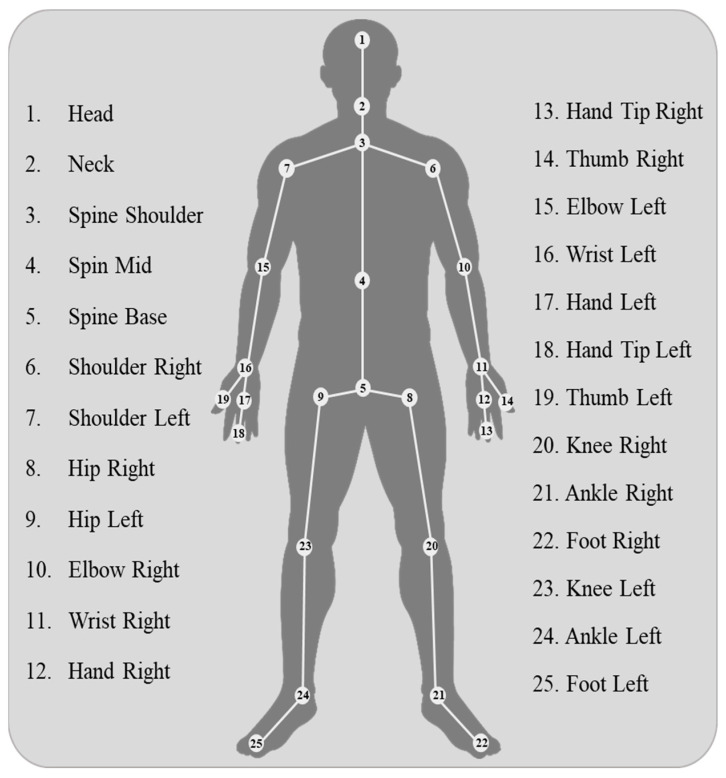
The human body skeleton joint extracted through Kinect.

In this study, we used two datasets for the PAR-Net evaluation. The first dataset, the Physical Activity Recognition Dataset (PAR) [36], is composed of 12 distinct activities performed by 20 different individuals aged 25–35 years. Data collection involved the use of Microsoft’s Kinect sensor V2, which is capable of extracting 25 different joints from the human body (refer to Figure 3). The dataset contains the x- and y-axis values of all body joints and stores them as CSV files. Each participant performed an activity for 10 s, resulting in 200 samples for each activity, with 10 repetitions per participant (totaling 120 samples per participant). The Kinect V2 sensor was employed to extract human skeleton joints using the Discrete Gestures Basics WPF SDK. Joint data were captured by the Kinect Body View script and saved as CSV files.

The data are organized in the sequence shown in Figure 3, representing 25 body joints, labeled numerically as indicated in Table 1. The labeled activity data files are consolidated and identified by their respective class numbers. All activity files are then merged into a single training file and transferred to the model using 5 different frame sequences, such as 30, 60, 90, 120, and 150 frame sequence data.

The second dataset used in this study is called the Physical Exercise Recognition Dataset (PER) [40], which is composed of 10 different poses that can be used to distinguish 5 exercise activities. The exercises are jumping jacks, push-ups, sit-ups, pull-ups, and squats. The dataset is composed of 33 different landmarks representing the positions of human body parts. A sequence of poses is provided to preserve the order of frames in each record. The data is collected from 447 videos in which different people are performing exercises, and these videos are collected from the Countix dataset, which provides the YouTube links to several activity videos. The videos are downloaded and preprocessed, where the frames are extracted from the videos and stored. They used the MediaPipe framework to extract the human skeletal data from the video frames, which can efficiently predict the location of 33 landmarks on the human body and face and store it as CSV files. The labels are provided with data in separate CSV files, which contain details about the data and their specific labels. The detailed explanation of both datasets used in this research is shown in Table 1.

### 4.2. Comparative Analysis of Different Models

**Figure 4:**
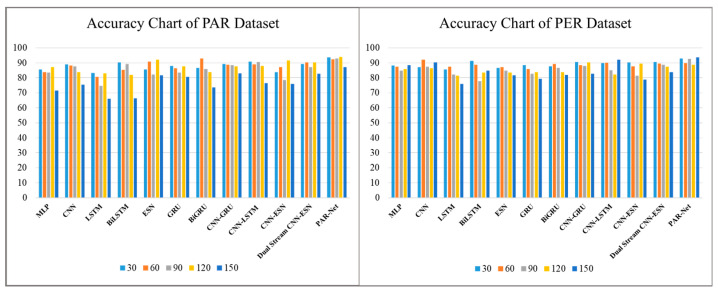
The accuracy of different models on the PAR and PER datasets.

The performance of different deep learning models is evaluated for activity recognition on different frame sequences, such as 30, 60, 90, 120, and 150 frames. The data of each frame are stored row-wise in a CSV file, where the information for each frame includes the coordinates of skeletal joints and corresponding labels indicating the specific activity. Therefore, each row of data represents one frame of the activity sequence, with the activity label in the last column. The term "frames" refers to the number of consecutive frames of skeleton data passed to the model as input. For example, when we mention using 30 frames of data, it means that we fed sequences of 30 consecutive frames of data to the model. The accuracy of each model is emphasized during these intervals, offering information about how effective it is in terms of accuracy, as reported in Table 2. The ablation results show a consistent tendency among the models evaluated, where most models show a reasonable level of accuracy at shorter intervals but are unable to sustain it as the frame intervals increase. Certain models, such as the MLP, CNN, LSTM, Bidirectional LSTM, ESN, GRU, BiGRU, CNN-GRU, CNN-LSTM, CNN–ESN, and Dual-Stream CNN–ESN without an attention mechanism, show good accuracy at certain intervals on both datasets but become less accurate at longer frames, suggesting that they are unable to capture long-term temporal dependencies. Nonetheless, the proposed dual-stream model, coupled with the attention mechanism, consistently obtains the best results comparatively. Figure 4 shows the accuracy of our proposed model compared to other models.

**Figure 5:**
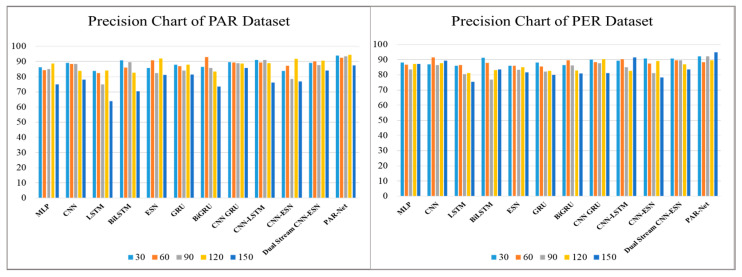
The precision of different models on the PAR and PER datasets.

**Figure 6:**
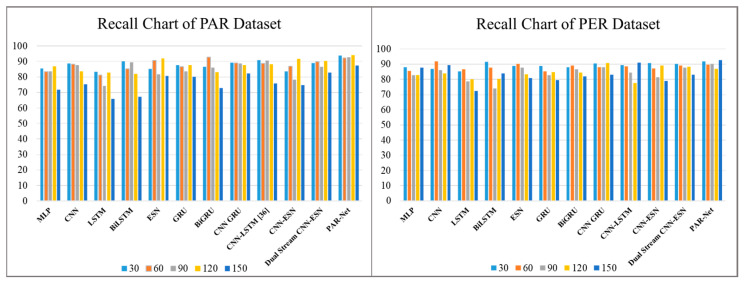
Recall comparisons of different models on the PAR and PER datasets.

**Figure 7:**
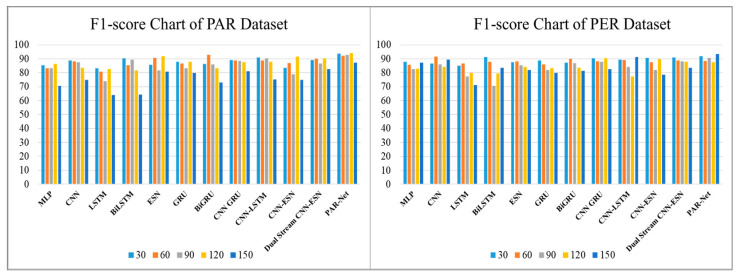
F1-score comparison of different models on the PAR and PER datasets.

Table 3 shows the precision scores generated by various deep-learning models used to recognize activities at different intervals of time: 30, 60, 90, 120, and 150 frames. These models include the MLP, CNN, LSTM, Bidirectional LSTM, ESN, GRU, BiGRU, CNN-GRU, CNN-LSTM, CNN–ESN, and Dual-Stream CNN–ESN without an attention mechanism. However, when the frame intervals increase, they show a reduction in accuracy, suggesting that greater periods cannot be reliably predicted. On the other hand, the PAR-Net consistently performs well with higher precision scores through all frame intervals and also maintains an impressive level of precision, peaking at 94.41% at 120 frames on the PAR dataset, while the peak precision score is 94.88% on the physical exercise recognition dataset. Such high performance highlights the PAR-Net's ability to reliably anticipate a wide range of actions, demonstrating its advantage over other models during evaluation across varying time intervals, as shown in Figure 5. The recall and F1-score metrics are also used to evaluate the performance of different models with diverse frame sequences, as shown in Table 4 and Table 5 respectively. The performance of these models decreases when the sequence increases. However, the PAR-Net performs well at longer intervals, peaking at 120 sequences with a recall value of 94.02% and a 94% F1-score on the physical activity recognition dataset, while the recall score and F1-score on the physical exercise dataset are 94.02% and 93.5%, respectively, as given in Figure 6 and Figure 7.

Overall, the PAR-Net performed better in all evaluated measures (accuracy, precision, recall, and F1-score), demonstrating its ability to capture complex temporal correlations and make accurate predictions over extended periods. Although some models demonstrate proficiency within particular temporal domains, they are unable to preserve stability and uniformity, particularly as the input sequences of the model increase. Therefore, the empirical results emphasize the PAR-Net as an optimal solution among the assessed models, supported by its dual-stream design and integrated attention mechanism. The ability of the PAR-Net to interpret activities over a range of time intervals highlights an optimal solution for activity recognition tasks, providing a thorough understanding of temporal and spatial dynamics in input data.

**Figure 8:**
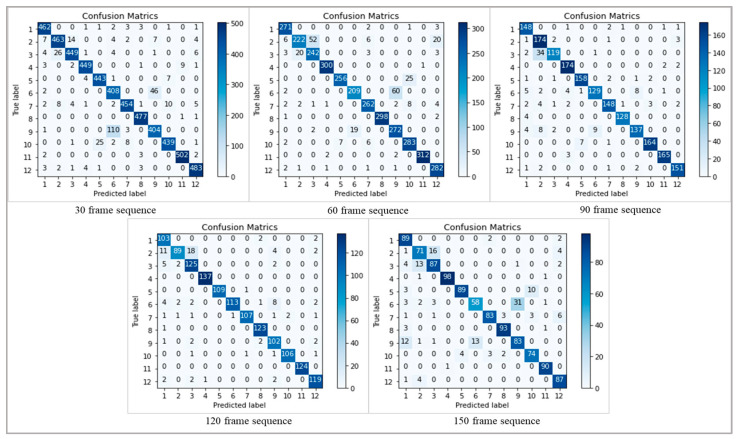
The confusion metrics of the PAR-Net on various sequences on the PAR dataset.

**Figure 9:**
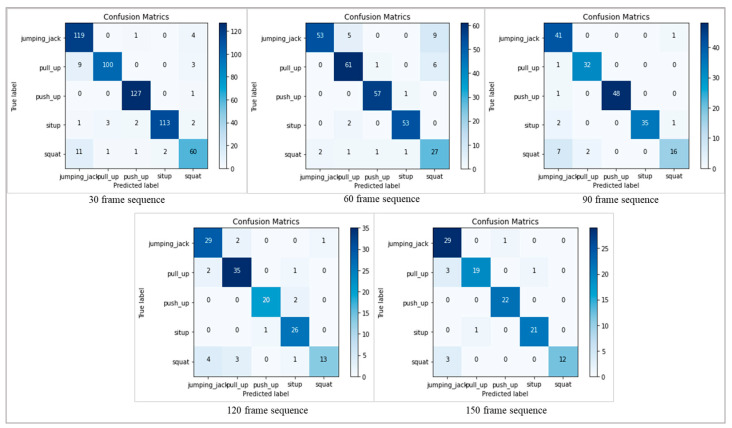
The confusion metrics of the PAR-Net on different sequences on the PER dataset.

In order to provide a thorough study of the model’s performance, we have included confusion matrices for both datasets at different frame sequences (30, 60, 90, 120, and 150). The confusion matrices, which provide the counts of true positive, true negative, false positive, and false negative predictions for each activity class, provide insightful information about the classification results. Figure 8 shows the confusion metrics of different frame sequences on the PAR dataset, where the activities in the confusion metrics are represented by the number sequences mentioned in Table 1. Figure 9 represents the confusion metrics of the PER dataset on various frame sequences.

### 4.3. Stability Analysis Test

**Figure 10:**
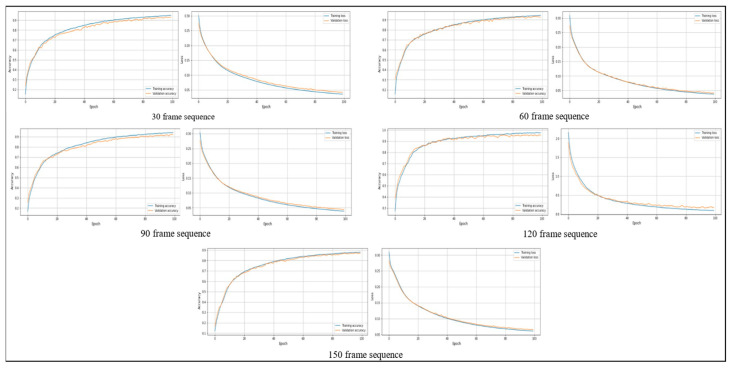
The training and validation accuracy and loss graphs on the PAR dataset.

**Figure 11:**
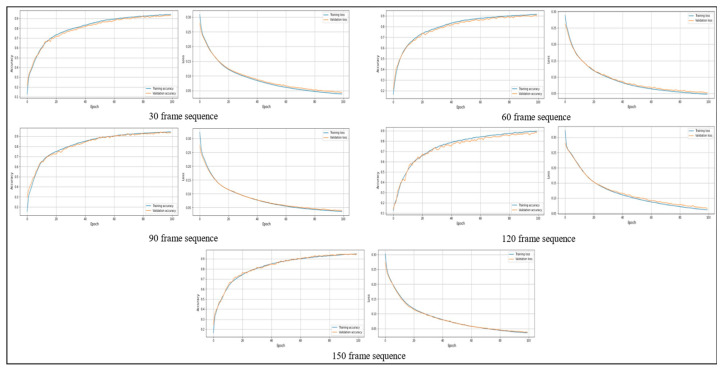
The training and validation accuracy and loss graphs on the PER dataset.

Stability analysis involves examining a model’s behavior and performance throughout many training iterations and epochs to determine its consistency and dependability. This research evaluates many aspects of the model’s learning dynamics, with a specific emphasis on its capacity to reach an optimum solution without experiencing overfitting or underfitting. Stability analysis involves examining the consistency and convergence of accuracy trends over consecutive epochs while analyzing the training and validation accuracy graphs. Both training and validation accuracy should ideally show consistent improvement and stability, indicating successful learning without affecting generalization. When analyzing the training and validation loss graphs, stability analysis entails observing the decreasing trend of loss values over epochs to verify that the model is efficiently reducing errors. An important part of stability analysis is recognizing the convergence of validation loss, which signals that the model has achieved its optimum performance on the validation set. Figure 10 and Figure 11 show the training and validation accuracy and loss on the PAR and PER datasets, respectively.

### 4.4. Statistical Significance Tests

Statistical significance tests, or *t*-tests, are used in our research to evaluate and guarantee the suggested model’s capacity for generalization. It is a systematic process used in statistical analysis to determine whether the results of the experiment provide strong enough evidence to reject a null hypothesis against an alternative hypothesis. This test aims to determine whether any differences or impacts in the data are statistically significant or just due to random variation. The procedure usually starts by creating two opposing hypotheses: the null hypothesis (H0), stating no impact or difference, and the alternative hypothesis (H1), proposing the existence of an effect. We chose a significance level (α) of 0.05 to determine the threshold of significance for approving or disapproving the null hypothesis. The significance test uses an appropriate test statistic, t-statistics, to produce either the *p*-value or critical value. The *p*-value indicates the likelihood of observing the data or more extreme data if the null hypothesis is true, but the critical value indicates the threshold at which the null hypothesis is rejected. The null hypothesis is determined by comparing the estimated *p*-value to the specified significance level or determining whether the test statistic is inside the rejection zone. When the *p*-value is less than α or when the test statistic exceeds the critical value, the null hypothesis is rejected, suggesting a statistically significant difference. If the *p*-value is greater than α or if the test statistic is within the non-rejection zone, the null hypothesis is not rejected since there is not enough evidence to support its rejection. The model is considered accurate if the t-statistic value is low, and the *p*-value is acquired through the comparison of the PAR-Net with another baseline model. The significance test provides a structured method to make meaningful inferences from data, facilitating informed decision-making and inference in scientific studies. Table 6 shows the significant test results comparison of the PAR-Net with other baseline models. 

The PAR-Net holds significant potential for real-world applications, particularly in healthcare, sports training, and smart home systems. In healthcare, it enables remote monitoring and customized therapies by tracking patients’ physical activity levels, which is especially beneficial for long-term health issues and rehabilitation. Athletes can benefit from improved training and performance monitoring through insights into movement patterns and workload management, reducing the risk of injuries. Integrating the model into smart home devices enhances comfort and energy efficiency by adjusting environmental parameters based on inhabitants’ activities and preferences. However, challenges arise from reliance on precise data collection devices and fluctuations in data integrity and external circumstances, necessitating improvements in data collection methods, noise handling, and validation through longitudinal studies. Investigating hybrid methodologies combining skeletal data with other modalities may enhance adaptability in various scenarios.

## 5. Conclusions and Future Work

This paper proposes a dual-stream architecture followed by an attention mechanism that can effectively recognize human physical activity. Using skeletal data and sophisticated models has been a key strategy in physical activity identification. By putting forth a novel solution, the PAR-Net performance is evaluated on two datasets aimed at transforming this field and efficiently recognizing human physical activity. The main goal is to recognize complex activity patterns across varied time intervals, broken down into frame sequences of 30, 60, 90, 120, and 150 sequences in both datasets, and compare the results of different methods. After a thorough analysis, the proposed model performs better than existing models. The PAR-Net demonstrated its superiority in properly identifying physical activities by outperforming other models over a wide range of temporal sequences.

### Limitations and Future Direction

The PAR-Net achieved higher performance; however, several limitations are associated with its current implementation and areas for future exploration. The PAR-Net's reliance solely on skeleton data disregards the potential information collected from other modalities like RGB images or depth maps. Incorporating multimodal data sources could enrich feature representation and boost the model's robustness. Furthermore, the model focuses on single-person skeletal data, which restricts its applicability in scenarios involving multiple individuals. Future research should prioritize developing models capable of concurrently processing multi-person skeletal data to effectively address real-world interactions. The PAR-Net uses multiple architectures to extract spatial and temporal features, which makes the model computationally expensive. Future research should explore the development of a unified architecture capable of extracting both spatial and temporal features efficiently for human physical activity recognition. This could involve the creation of a single architecture tailored to handle both types of features, potentially reducing computational complexity while maintaining performance. Moreover, real-time deployment considerations require optimization of the model architecture and inference techniques for efficient real-time performance. Furthermore, the absence of a comprehensive graphical user interface (GUI) hinders the model's accessibility and usability, highlighting the need for an intuitive interface design. By addressing these limitations and exploring potential avenues for enhancement, we aim to advance the efficacy and applicability of human physical activity recognition systems.

## Figures and Tables

**Table 1:** Types of activities and skeleton points in each dataset.

Datasets	Skeleton Joints Data			Activities
PAR Dataset	1. Head 2. Neck 3. Spine Shoulder 4. Spin Mid 5. Spine Base 6. Shoulder Right 7. Shoulder Left 8. Hip Right 9. Hip Left	10. Elbow Right 11. Wrist Right 12. Hand Right 13. Hand Tip Right 14. Thumb Right 15. Elbow Left 16. Wrist Left 17. Hand Left	18. Hand Tip Left 19. Thumb Left 20. Knee Right 21. Ankle Right 22. Foot Right 23. Knee Left 24. Ankle Left 25. Foot Left	1. Overhead Arm Raise 2. Front Arm Raise 3. Arm Curl 4. Chair Stand 5. Balance Walk 6. Side Leg Raise (Right, Left) 7. Shoulder 8. Chest 9. Leg Raise (Forward, Backward) 10. Arm Circle 11. Side Twist (Right, Left) 12. Squats
PER Dataset	0. nose 1. left_eye_inner 2. left_eye 3. left_eye_outer 4. right_eye_inner 5. right_eye 6. right_eye_outer 7. left_ear 8. right_ear 9. mouth_left 10. mouth_right	11. left_shoulder 12. right_shoulder 13. left_elbow 14. right_elbow 15. left wrist 16. right_wrist 17. left_pinky 18. right_pinky 19. left_index 20. right_index 21. left_thumb	22. right_thumb 23. left_hip 24.right_hip 25. left_knee 26. right_knee 27. left_ankle 28. right_ankle 29. left_heel 30. right_heel 31. left_foot_index 32. right_foot_index	1. Jumping Jack 2. Push-ups 3. Pull-ups 4. Sit-ups 5. Squats

**Table 2:** Accuracy comparison of models across different frames.

PAR Dataset						
No.	Model Name	30	60	90	120	150
1	MLP	85.45	83.64	83.47	87.05	71.51
2	CNN	88.88	88.22	87.65	83.74	75.47
3	LSTM	83.31	80.64	74.69	82.92	66.09
4	BiLSTM	90.15	85.39	89.30	82.02	66.26
5	ESN	85.55	90.73	82.17	92.01	81.66
6	GRU	87.96	86.32	83.57	87.74	80.63
7	BiGRU	86.57	92.83	85.89	83.74	73.49
8	CNN-GRU	89.25	88.63	88.48	87.60	82.96
9	CNN-LSTM [36]	90.89	88.98	90.44	87.94	76.50
10	CNN–ESN	83.76	87.01	78.56	91.66	75.81
11	Dual-Stream CNN–ESN	89.10	90.15	87.03	90.28	82.78
12	PAR-Net	93.69	92.33	92.82	93.93	87.09
PER Dataset						
1	MLP	88.21	87.50	84.82	85.71	88.39
2	CNN	87.14	92.14	87.50	86.42	90.17
3	LSTM	85.53	87.50	82.14	81.42	75.89
4	BiLSTM	91.42	88.57	77.67	83.57	84.82
5	ESN	86.67	87.25	84.82	83.52	81.77
6	GRU	88.56	85.90	82.70	83.75	79.35
7	BiGRU	87.66	89.20	86.50	83.78	81.89
8	CNN-GRU	90.57	88.55	87.99	90.33	82.66
9	CNN-LSTM	89.64	90.00	85.00	82.14	91.96
10	CNN–ESN	90.17	87.65	81.53	89.54	78.82
11	Dual-Stream CNN–ESN	90.50	89.54	88.78	87.47	83.78
12	PAR-Net	92.85	89.64	92.51	88.57	93.75

**Table 3:** Precision comparison of models across different frames.

PAR Dataset						
No.	Model Name	30	60	90	120	150
1	MLP	86.18	84.37	85.12	88.54	74.97
2	CNN	89.20	88.48	88.37	83.93	78.04
3	LSTM	83.94	82.51	74.95	84.04	64.01
4	BiLSTM	90.74	85.90	89.62	82.52	70.35
5	ESN	85.81	90.77	82.37	92.03	81.10
6	GRU	87.91	86.95	84.02	87.89	81.51
7	BiGRU	86.58	92.95	85.80	83.30	73.48
8	CNN GRU	89.49	89.28	88.79	88.62	85.87
9	CNN-LSTM [36]	91.11	89.31	91.13	88.82	76.13
10	CNN–ESN	83.92	87.23	78.56	91.78	76.85
11	Dual-Stream CNN–ESN	89.22	90.20	87.62	90.52	84.02
12	PAR-Net	93.84	92.53	93.48	94.41	87.39
PER Dataset						
1	MLP	88.08	86.65	83.61	87.29	87.19
2	CNN	86.90	91.53	86.55	87.61	89.37
3	LSTM	85.99	86.59	80.48	81.08	75.53
4	BiLSTM	91.27	87.85	76.85	83.22	83.68
5	ESN	86.07	86.07	83.33	85.11	81.55
6	GRU	88.10	85.60	82.20	82.55	80.10
7	BiGRU	86.56	89.55	86.21	82.90	80.98
8	CNN GRU	90.10	88.29	87.56	90.25	81.25
9	CNN-LSTM	89.43	90.23	85.09	82.58	91.51
10	CNN–ESN	90.85	87.39	81.22	89.21	78.22
11	Dual-Stream CNN–ESN	90.90	89.70	89.54	86.95	83.66
12	PAR-Net	92.31	88.40	92.23	89.57	94.88

**Table 4:** Recall comparison of models across different frames.

PAR Dataset						
No.	Model Name	30	60	90	120	150
1	MLP	85.39	83.43	83.58	86.86	71.92
2	CNN	88.86	88.07	87.77	83.50	75.36
3	LSTM	83.24	81.23	74.15	82.84	65.89
4	BiLSTM	90.05	85.24	89.41	82.11	67.16
5	ESN	85.22	90.64	81.73	92.03	80.77
6	GRU	87.70	86.63	83.64	87.78	80.24
7	BiGRU	86.46	92.85	86.01	83.19	72.87
8	CNN GRU	89.22	89.09	88.61	87.58	82.32
9	CNN-LSTM [36]	90.84	88.79	90.56	88.10	75.82
10	CNN–ESN	83.57	86.97	78.15	91.64	74.66
11	Dual-Stream CNN–ESN	88.99	89.92	86.71	90.34	82.75
12	PAR-Net	93.78	92.25	92.70	94.02	87.29
PER Dataset						
1	MLP	87.99	85.60	82.72	82.75	87.63
2	CNN	86.93	91.78	86.13	83.82	89.48
3	LSTM	85.15	86.75	78.76	80.03	72.39
4	BiLSTM	91.46	87.71	74.08	80.34	84.05
5	ESN	88.98	90.13	87.72	83.33	81.03
6	GRU	88.90	85.15	82.87	84.65	79.55
7	BiGRU	88.10	89.23	86.78	84.55	82.10
8	CNN GRU	90.55	87.99	88.12	90.74	83.14
9	CNN-LSTM	89.40	88.65	84.59	77.61	91.06
10	CNN–ESN	90.89	87.15	81.36	89.14	78.87
11	Dual-Stream CNN–ESN	90.35	89.12	87.85	88.20	83.22
12	PAR-Net	91.75	89.56	90.25	86.89	92.68

**Table 5:** F1-score comparison of models across different frames.

PAR Dataset						
No.	Model Name	30	60	90	120	150
1	MLP	85.37	83.32	83.12	86.36	70.61
2	CNN	88.82	88.10	87.46	83.37	74.74
3	LSTM	83.08	80.68	73.98	82.47	63.87
4	BiLSTM	90.15	85.43	89.43	81.76	64.45
5	ESN	85.55	90.66	81.53	91.96	80.66
6	GRU	87.69	86.57	83.29	87.76	79.91
7	BiGRU	86.39	92.86	85.82	83.03	72.88
8	CNN GRU	89.17	88.74	88.59	87.44	80.96
9	CNN-LSTM [36]	90.85	88.87	90.29	87.80	75.13
10	CNN–ESN	83.56	86.94	78.89	91.63	74.81
11	Dual-Stream CNN–ESN	88.97	89.92	86.58	90.22	82.57
12	PAR-Net	93.73	92.18	92.90	94.00	87.18
PER Dataset						
1	MLP	87.82	85.67	82.52	82.76	87.28
2	CNN	86.64	91.62	85.93	84.22	89.24
3	LSTM	84.99	86.61	77.47	79.77	71.12
4	BiLSTM	91.33	87.74	70.46	79.43	83.44
5	ESN	87.50	88.05	85.47	84.21	82.10
6	GRU	88.70	86.10	82.10	83.10	79.87
7	BiGRU	87.10	89.89	86.89	83.65	81.25
8	CNN GRU	90.22	88.25	87.88	90.35	82.54
9	CNN-LSTM	89.34	89.04	84.22	77.29	91.18
10	CNN–ESN	90.66	87.58	81.87	89.55	78.55
11	Dual-Stream CNN–ESN	90.88	88.90	88.22	87.77	83.50
12	PAR-Net	91.94	88.54	90.70	87.51	93.50

**Table 6:** Significance test results for comparative analysis of the PAR-Net with other baselines using *p*-values.

No.	Model Name	*p*-Value
1	PAR-Net vs. MLP	0.015
2	PAR-Net vs. CNN	0.029
3	PAR-Net vs. LSTM	0.025
4	PAR-Net vs. BiLSTM	0.030
5	PAR-Net vs. ESN	0.029
6	PAR-Net vs. GRU	0.028
7	PAR-Net vs. BiGRU	0.029
8	PAR-Net vs. CNN-GRU	0.033
9	PAR-Net vs. CNN-LSTM	0.034
10	PAR-Net vs. CNN–ESN	0.036
11	PAR-Net vs. Dual-Stream CNN–ESN	0.041

## Data Availability

Data are contained in this article.
